# Interactions across emotional, cognitive and subcortical motor networks underlying freezing of gait

**DOI:** 10.1016/j.nicl.2023.103342

**Published:** 2023-02-02

**Authors:** Hiroki Togo, Tatsuhiro Nakamura, Noritaka Wakasugi, Yuji Takahashi, Takashi Hanakawa

**Affiliations:** a Department of Integrated Neuroanatomy and Neuroimaging, Kyoto University Graduate School of Medicine, Kyoto, Yoshida-Konoe, Sakyo-ku, Kyoto 606-8501, Japan; b Department of Advanced Neuroimaging, Integrative Brain Imaging Center, National Center of Neurology and Psychiatry (NCNP), 4-1-1, Ogawa-Higashi, Kodaira, Tokyo 187-8551, Japan; c Department of Neurology, National Center Hospital, National Center of Neurology and Psychiatry (NCNP), Tokyo, 4-1-1, Ogawa-Higashi, Kodaira, Tokyo 187-8551, Japan

**Keywords:** FOG, Freezing of gait, PD, Parkinson’s disease, HC, healthy control, rsfcMRI, Resting-state functional connectivity MRI, FC, functional connectivity, RSN, resting-state network, SMN, sensorimotor network, CBLN, cerebellar network, BGN, basal basal ganglia network, FPN, frontoparietal network, SMA, supplementary motor area, MFG, middle frontal gyrus, IFG, inferior frontal gyrus, OFC, orbitofrontal cortical region, ACC, anterior cingulate cortex, AG, angular gyrus, SMG, supramarginal gyrus, PT, planum temporale, PAG, periaqueductal gray matter, NFOGQ, new freezing of gait questionnaire, MMSE, Mini-Mental State Examination, MDS-UPDRS-III, Unified Parkinson’s Disease Rating Scale Part III, LED, levodopa equivalent daily dose, ICA, independent component analysis, FWE, familywise error, LASSO, least absolute shrinkage and selection operator, Freezing of gait, Parkinson’s disease, Resting state fMRI, Functional connectivity

## Abstract

Freezing of gait (FOG) is a gait disorder affecting patients with Parkinson’s disease (PD) and related disorders. The pathophysiology of FOG is unclear because of its phenomenological complexity involving motor, cognitive, and emotional aspects of behavior. Here we used resting-state functional MRI to retrieve functional connectivity (FC) correlated with the New FOG questionnaire (NFOGQ) reflecting severity of FOG in 67 patients with PD. NFOGQ scores were correlated with FCs in the extended basal ganglia network (BGN) involving the striatum and amygdala, and in the extra-cerebellum network (CBLN) involving the frontoparietal network (FPN). These FCs represented interactions across the emotional (amygdala), subcortical motor (BGN and CBLN), and cognitive networks (FPN). Using these FCs as features, we constructed statistical models that explained 40% of the inter-individual variances of FOG severity and that discriminated between PD patients with and without FOG. The amygdala, which connects to the subcortical motor (BGN and CBLN) and cognitive (FPN) networks, may have a pivotal role in interactions across the emotional, cognitive, and subcortical motor networks. Future refinement of the machine learning-based classifier using FCs may clarify the complex pathophysiology of FOG further and help diagnose and evaluate FOG in clinical settings.

## Highlight

• The interactions across resting-state networks underlie freezing of gait (FOG).
• The amygdala (emotional network) connects to the motor and cognitive networks.
• Machine learning analysis from connectivities revealed the pathophysiology of FOG.

## 1 Introduction

Freezing of gait (FOG), defined as “brief, episodic absence or marked reduction of forward progression of the feet despite the intention to walk” (Nutt et al., 2011), is a gait disorder affecting those with advanced Parkinson’s disease (PD) and related disorders. FOG may result in a fall, thereby inducing a fear of falling (Ghielen et al., 2020) and reducing the patient’s quality of life (Moore et al., 2007, Perez-Lloret et al., 2014). It is challenging to develop effective therapies partly because the phenomenology and pathophysiology of FOG are complicated.

Problems in attentional/behavioral “set-shift” (Ehgoetz Martens et al., 2016, Smulders et al., 2015) and cognitive/executive functions (Brugger et al., 2015, Naismith et al., 2010) underlie FOG in addition to the impaired motor basal-ganglia circuits that underlie parkinsonian gait (Hanakawa et al., 1999b, Hanakawa, 2006). Moreover, anxiety often provokes FOG (Ehgoetz Martens et al., 2014, Ehgoetz Martens et al., 2015). Therefore, the interactions between the motor, cognitive, and emotional circuits are likely to be key in understanding the pathophysiology underlying FOG (Lewis and Barker, 2009).

Previous task functional magnetic resonance image (fMRI) studies on FOG used gait imagery (Snijders et al., 2011) or virtual reality tasks (Ehgoetz Martens et al., 2018, Shine et al., 2013a, Shine et al., 2013b), which yielded somewhat different results: the activation in the supplementary motor area (SMA) (Snijders et al., 2011) or the mesencephalic locomotor region (Shine et al., 2013a, Snijders et al., 2011), the functional connectivity between cognitive control and basal ganglia network (BGN) (Shine et al., 2013b), and the involvement of the emotional network (Ehgoetz Martens et al., 2018). In addition to brain activity, it is imperative to understand the brain connectivity underlying pathophysiological changes (Bharti et al., 2019b, Fasano et al., 2015). Resting-state functional connectivity MRI (rsfcMRI) measures functional connectivity (FC) over a dozen resting-state networks (RSNs) (Fox et al., 2007, Smith et al., 2009), providing useful markers to help evaluate neuropsychiatric disorders (Takamura and Hanakawa, 2017). However, the findings of previous rsfcMRI studies on FOG were inconsistent, reporting various patterns of alterations of FCs in the sensorimotor network (SMN) (Canu et al., 2015), cerebellar network (CBLN) (Bharti et al., 2019a, Fasano et al., 2017), CBLN-basal basal ganglia network (BGN) (Bharti et al., 2019a, Fasano et al., 2017), or the SMA-mesencephalic/cerebellar locomotor region network (Fling et al., 2014). This inconsistency may be partly related to the limited sample sizes (n < 40) in the previous studies (Bharti et al., 2019a, Canu et al., 2015, Fling et al., 2014, Zhou et al., 2018). Moreover, most previous studies on FOG focused only on one of the motor, cognitive, or emotional networks, and thus may not have fully characterized the system-level impairment.

A previous fMRI study with a virtual reality task showed the association of task-induced FCs across motor, cognitive, and emotional regions with the “freezing index” integrating motor, cognitive, and emotional neuropsychological scores (Ehgoetz Martens et al., 2018). A previous rsfcMRI study with a volume-of-interest (VOI)-based approach showed abnormal FC between the emotional network (amygdala VOI) and the putaminal VOI, and between the amygdala VOI and the cognitive/attentional frontoparietal network (FPN) in patients with FOG (Gilat et al., 2018). However, this study focused on the influence of emotional networks on the motor or cognitive networks. Therefore, the role of interactions across the motor, cognitive, and emotional networks in FOG is yet to be shown using rsfcMRI data. Moreover, it would enhance our system-level understanding of FOG pathophysiology if a statistical model based on rsfcMRI and machine learning technology was developed to predict the severity of FOG.

The primary research objective was to build a computational model that can predict FOG severity from rsfcMRI to help understand the interactions across the motor, cognitive, and emotional networks underlying FOG. Such a model was expected to characterize the system-level impairment underlying FOG and to improve our understanding of its pathophysiology. To this end, we first analyzed rsfcMRI data from PD patients and healthy controls to retrieve reliable sets of RSNs. Using a dual regression approach that elucidated intra- and extra-network connectivities, we then analyzed the gait-related RSNs (BGN, CBLN, and SMN), assuming altered FC within the gait-related motor RSNs as well as across the motor, emotional, and cognitive RSNs. These findings enabled us to construct a statistical model that explained the inter-individual variability of FOG severity based on that of FCs across motor, cognitive, and emotional RSNs.

## 2 Material and methods

### 2.1 Participants

Seventy-one people with PD (mean age: 68.4 years, standard deviation: 8.0 years, 43 males/28 females) were recruited. Each participant provided written informed consent to participate in the study. The inclusion criteria were defined according to the UK Parkinson’s Disease Society Brain Bank clinical diagnostic criteria (Hughes et al., 1992). The exclusion criteria were: (1) contraindications to MRI and (2) local brain lesions (e.g., brain tumor or cerebral infarction) incidentally identified on MRI. Data from 57 age-matched healthy controls (HC) (mean age: 69.5 years, standard deviation: 6.5 years, 36 males/21 females) from the institute’s rsfcMRI database were also used. MRI scans of PDs and HCs were acquired using the same scanner and imaging protocol. The study protocol was approved by the Ethics Committee of the National Center of Neurology and Psychiatry, Tokyo, Japan (A2019-126).

### 2.2 Data acquisition

#### 2.2.1 Clinical and neuropsychological assessment

The severity of FOG was evaluated using the new freezing of gait questionnaire (NFOGQ), with scores ranging from 0 to 28 (high scores indicated more severe FOG) (Nieuwboer et al., 2009). The NFOGQ was provided to participants within 2 weeks before or after the MRI acquisition. The Movement Disorder Society-sponsored revision of the Unified Parkinson’s Disease Rating Scale Part III (MDS-UPDRS-III) and Mini-Mental State Examination score (MMSE) were acquired by neurologists. All evaluation data were collected at the medication-on status. The levodopa equivalent daily dose (LED) was calculated (Tomlinson et al., 2010).

#### 2.2.2 MRI data acquisition

All patients were scanned at the medication-on state. The rsfcMRI data were acquired on a 3-T MRI scanner (Siemens, MAGNETOM Verio Dot) using a 32-channel phased-array head coil. Foam cushions and earplugs were used to limit head motion and reduce scanner noise, respectively. RsfcMRI scans were acquired using a gradient-echo, echo-planar imaging sequence with a repetition time (TR) of 2500 ms, echo time (TE) of 30 ms, a flip angle of 80°, and 49 axial slices with the posterior-anterior phase encoding direction, which yielded a 3.3 × 3.3 × 4.0 (0.8-mm interslice gaps) mm^3^ voxel size. All participants underwent a 10-min rsfcMRI scan with their eyes open and fixating on a crosshair; they were instructed to remain awake and not to think of anything specific. Field map MRI was acquired with a double-echo spoiled gradient-echo sequence (TR = 488.0 ms, TE = 4.92/7.38 ms, voxel size = 3.3 × 3.3 × 4.0 (0.8-mm gaps) mm^3^, flip angle = 60°), and whole-brain three-dimensional T1-weighted MRI was performed with a magnetization prepared rapid gradient echo (MP-RAGE) sequence (TR = 1900 ms, TE = 2.52 ms, inversion time (TI) = 900 ms, flip angle = 9°, 192 sagittal slices, and voxel size = 1.00 × 0.98 × 0.98 mm^3^).

### 2.3 Imaging data preprocessing

Non-brain tissues and cerebrospinal fluid were removed from structural MRI, using the SPM12 software (https://www.fil.ion.ucl.ac.uk/spm/software/spm12/). After deleting the first three volumes, rsfcMRI data were preprocessed using FSL (FMRIB’s Software Library, https://www.fmrib.ox.ac.uk/fsl), including field-map distortion correction (Togo et al., 2017) and motion correction. We performed an initial data quality check (especially in terms of head motion) separately for translation and rotation parameters using the following formula:

$$\frac{1}{M-1}\sum_{i=2}^{M}\sqrt{{\left|x_{i}-x_{i-1}\right|}^{2}+{\left|y_{i}-y_{i-1}\right|}^{2}+{\left|z_{i}-z_{i-1}\right|}^{2}}$$

where M is the total number of time points, and *x_i_*, *y_i_*, and *z_i_* are translations or rotations in the three axes at time point *i,* calculated with FEAT in the preprocessing step. Four subjects were excluded from further analysis because of excessive head motion (i.e., translation > 0.3 mm or rotation > 0.3°) (Liu et al., 2008). Finally, we analyzed data from 67 people with PD. Non-brain tissue removal of the rsfcMRI data were followed by spatial smoothing (6-mm full-width-at-half-maximum Gaussian kernel), and high-pass temporal filtering with a cutoff frequency of 0.01 Hz. Single-session independent component analysis (ICA) was performed using Multivariate Exploratory Linear Optimized Decomposition into Independent Components. Autoclassification of artifactual ICA spatial components was performed using the ICA-based Xnoiseifier (Salimi-khorshidi et al., 2014); the noise components were regressed out from the data. The noise-cleaned rsfcMRI data were registered to the individual structural images using boundary-based registration (Greve and Fischl, 2009) and then to the Montreal Neurological Institute template using nonlinear registration with FMRIB’s Nonlinear Image Registration Tool, before being resampled to 4-mm isovoxels.

### 2.4 Component identification and statistics

**Fig. 1:**
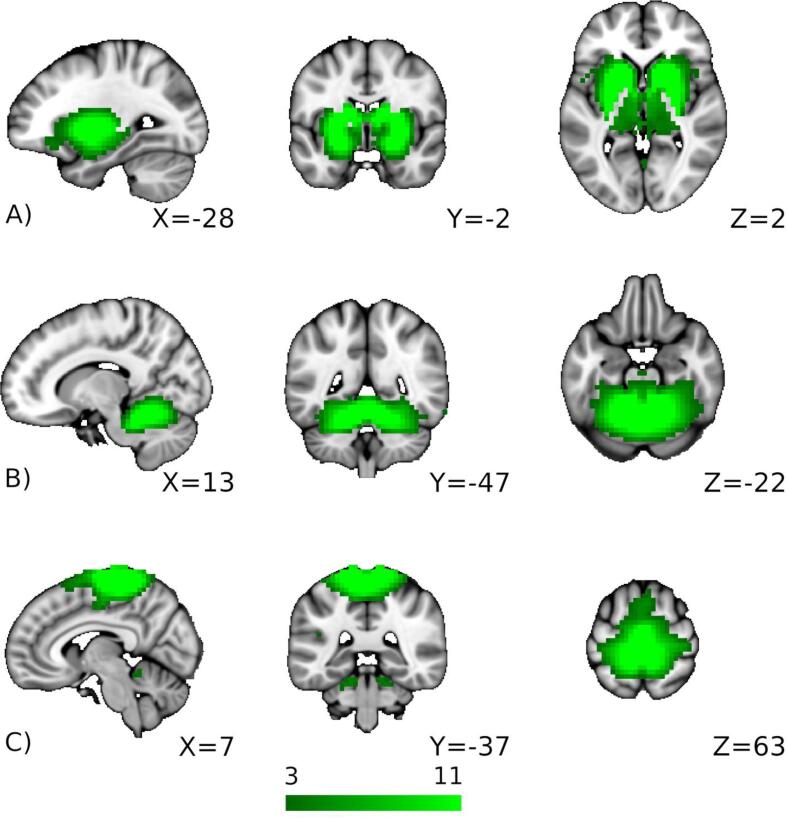
Three resting-state networks of interest (green). A) Basal ganglia network (BGN), B) cerebellar network (CBLN), and C) sensorimotor network (SMN). The color bar indicates z-values thresholded at the default value (z > 3). (For interpretation of the references to color in this figure legend, the reader is referred to the web version of this article.)

Group-spatial ICA was conducted on the rsfcMRI data from the 67 PDs and 57 HCs to detect the general and pathophysiological RSNs (Griffanti et al., 2016). The concatenated rsfcMRI volumes were decomposed into 40 spatial components. We visually investigated all ICA components and identified the three ICA components of interest according to the previous literature BGN (Shine et al., 2013b, Szewczyk-Krolikowski et al., 2014), CBLN (Fasano et al., 2017, Fling et al., 2014, Jahn et al., 2008), and SMN (Shine et al., 2013b) (Fig. 1). We also performed group-spatial ICA on the rsfcMRI data only from the 67 PDs for a reference (Supplementary Fig. 1).

### 2.5 Dual regression analysis

In the PD patients, all 40 spatial maps from the group ICA were used to generate participant-specific versions of the spatial maps and associated time series using the dual regression approach (Filippini et al., 2009, Nickerson et al., 2017). First, the RSN template extracted by group-ICA was used as the first regressor, and time-series data for each subject were extracted from the rs-fMRI data. Next, using this time-series data as the regressor, we extracted a spatial map (RSNs) for each subject. We then performed a linear regression analysis to investigate the correlation between the NFOGQ scores and FC related to the three RSNs of interest, using Permutation Analysis of Linear Models (PALM). PALM provided nonparametric familywise error (FWE) correction over the multiple voxels and the number of RSNs of interest simultaneously (Winkler et al., 2014, Winkler et al., 2016). We ran a permutation 5000 times in PALM. We also performed the FC analysis including the age and sex as covariates, resulting in similar clusters (see Supplementary Materials).

### 2.6 Statistics and prediction model

We conducted Spearman’s tests to test the correlations between NFOGQ scores and MDS-UPDRS-III, disease duration, LED, or MMSE at a threshold of *p* < 0.05. In the PALM analysis of FC, we used a threshold of *p* < 0.05 corrected for FWE using threshold-free cluster enhancement (Smith and Nichols, 2009).

We also constructed a statistical model that explained NFOGQ scores from FC, using least absolute shrinkage and selection operator (LASSO). First, we extracted signal time-courses from volumes-of-interest (VOIs) placed in the rsfcMRI data. According to the results from the dual regression analysis below, we extracted noise-cleaned signal time-courses from two RSNs (BGN and CBLN), four BGN-related intra-network VOIs (right amygdala, left inferior and superior striatum, and right striatum) and seven CBLN-related extra-network VOIs (left middle frontal gyrus (MFG), left inferior frontal gyrus (IFG), left orbitofrontal cortical region (OFC), right anterior cingulate cortex (ACC), right angular gyrus (AG), right supramarginal gyrus (SMG), and planum temporale (PT)). Pairwise correlations among the extracted time-courses yielded 77 feature values, which were fed to the LASSO models for predicting NFOGQ scores. A feature value (right amygdala-right striatum FC), which was correlated with LED, was excluded from the LASSO models. We performed the feature extraction through stability selection and LASSO linear regression using the following formula.

$${\hat{\beta }}^{\lambda }=argmin\left\{{\left\|Y-X\beta \right\|}_{2}^{2}+\lambda \sum_{k=1}^{p}\frac{\left|\beta_{k}\right|}{s_{k}}\right\}\left(s_{k}\;\left[\alpha ,1\right],0<\alpha <1\right)$$

We used five-fold nested cross validation to tune the hyper-parameter (λ) and to estimate R^2^ values and root mean squared error (RMSE) of the model for predicting the FOGQ scores. We repeated the cross validation fifty times and averaged the R^2^ values and the RMSE to evaluate the model precision. The FCs that selected>50 % of the repetitions were adopted as relevant features. We also constructed a sparse logistic regression model using the feature values selected by the LASSO to discriminate between patients with FOG and those without FOG. Again, we used stratified fivefold nested cross validation to tune the hyper-parameter (λ) and to estimate the balanced accuracy (the average of the sensitivity and specificity), and the sensitivity and specificity of the model (the proportion of the positive cases were maintained). For operational binary classification, patients with an NFOGQ score > 0 were defined as FOG positive (FOG+) and those with a null NFOGQ score were defined as FOG negative (FOG−). Using these criteria, the patients were divided into 47 cases with FOG (FOG+) and 20 cases without FOG (FOG−). We simulated a binomial distribution of the same number of subjects (n = 67) to obtain a statistical inference of the estimated balanced accuracy, and the sensitivity and specificity of the model (*p* < 0.05). Also, we calculated the area under the curve (AUC).

## 3 Results

### 3.1 Demographics

The NFOGQ scores were correlated with MDS-UPDRS-III and LED (Table 1). Twenty-nine patients had higher MDS- UPDRS-III scores on the left side and 21 patients on the right side. In the remaining 10 patients, there was no asymmetry or the information was missing.

**Table 1:** Demographic information of study participants.

		Correlation coefficient
NFOGQ	10.3 (8.6)	
Sex (M/F)	40/27	
Age (years)	68.5 (8.2)	
Disease duration (years)	7.5 (4.7)	0.165
MDS-UPDRS-III (n = 60)^§^	26.8 (14.5)	0.2582**
LED (mg/day)	631.7 (422.1)	0.308*
MMSE	27.6 (3.2)	−0.140

* p < 0.05, ** p < 0.01 by Spearman’s rank order correlation. LED = levodopa equivalent daily dose, MMSE = mini-mental state examination score, MDS-UPDRS-III = The Movement Disorder Society-sponsored revision of the Unified Parkinson’s Disease Rating Scale Part III, M = male, F = female.

^§^MDS-UPDRS-III was not available for seven patients.

### 3.2 Functional connectivity

**Fig. 2:**
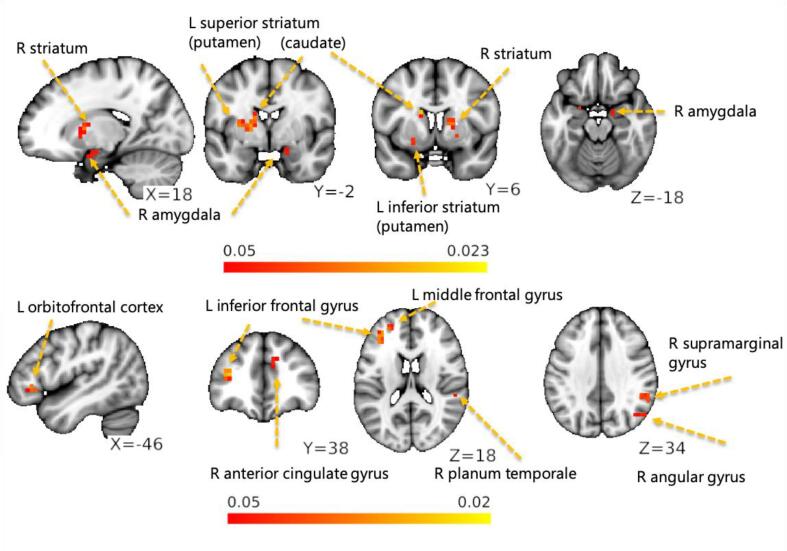
The hot color scaled areas indicate a correlation of freezing severity with functional connectivity of the basal ganglia network (top) and cerebellar network (bottom) (corrected *p* < 0.05).

We tested the correlation of the NFOGQ scores with the intra- and extra-FC attached to the three RSNs (BGN, CBLN, and SMN). The NFOGQ scores were positively correlated with the clusters associated with the BGN and CBLN (Fig. 2). The NFOGQ-correlated BGN clusters were found within the BGN mask (intra-RSN), including the caudate nucleus, putamen, and amygdala. The NFOGQ-correlated CBLN clusters were found outside the CBLN mask (extra-RSN) including the left MFG, left OFC, parts of the FPN (left IFG, right AG, and right SMG), and right ACC. The SMN did not reveal any clusters correlated with the NFOGQ scores. These findings were replicated with and without using age and sex as covariates (supplementary Fig. 2). However, we failed to find clusters correlated with the NFOGQ scores when we used the three RSNs of interest created from the group ICA of the 67 PDs only.

### 3.3 Statistical model using FCs to predict NFOGQ scores and the presence of FOG

**Fig. 3:**
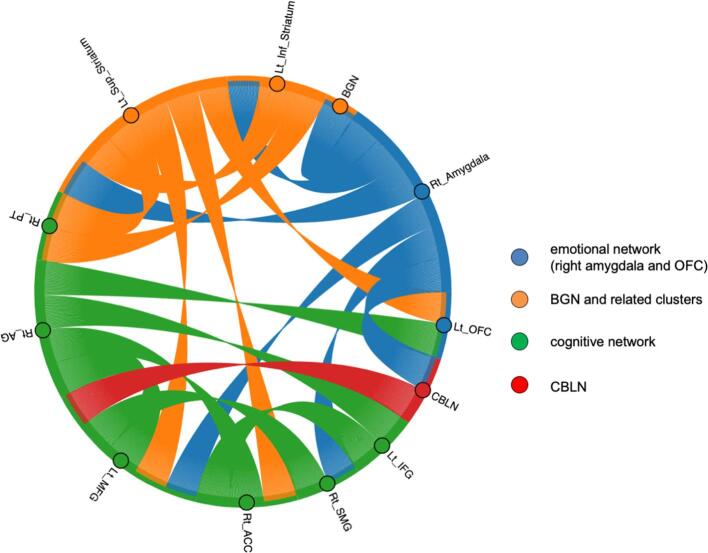
A chord diagram of the 19 FCs used to predict NFOGQ scores. The circles indicate the 12 nodes (brain regions and RSNs): blue represents emotional regions (right amygdala and OFC), orange represents the basal ganglia network (BGN) and related clusters, red represents the cerebellar network (CBLN), and green represents cognitive regions. The lines indicate the edges: blue represents connectivity involving the amygdala, orange represents connectivity involving BGN, red represents connectivity involving the CBLN, and green represents connectivity across the cognitive regions. (For interpretation of the references to color in this figure legend, the reader is referred to the web version of this article.)

To construct a statistical model explaining NFOGQ with FCs, we investigated 77 FCs derived from all NFOGQ-correlated nodes and the three RSNs of original interest. Among them, the FC between the right amygdala and right striatal nodes was removed from the following model because it also correlated with LED, making the interpretation difficult (Tahmasian et al., 2015). We found that the LASSO model consisted of 19 FCs (Table 2) that best predicted the NFOGQ scores (estimated R^2^; mean: 0.407, standard deviation: 0.25, RMSE; mean: 6.295, standard deviation: 0.92; Fig. 3). Four nodes were associated with the BGNs and seven nodes with the CBLNs. The nodes included the emotional network (amygdala and left OFC), subcortical motor network (superior and inferior striatal regions and CBLN), and cognitive network (left IFG, right AG, right SMG, and right PT). Notably, 6 out of 19 FCs included the amygdala, which connects to the subcortical motor (BGN and CBLN) and cognitive (FPN) networks. Other FCs included the intra-BGN, striatum-FPN (four FCs), striatum-OFC, intra-FPN (four FCs), FPN-OFC (two FCs), and the CBLN-FPN.

**Table 2:** Combinations of FCs used in the model to predict NFOGQ scores. estimated R^2^; mean: 0.407, standard deviation: 0.25. FCs = functional connectivities.

Combinations of FC	beta value
Right amygdala - basal ganglia network (BGN)	−2.3 ± 2.8
Right amygdala - cerebellum network (CBLN)	−5.1 ± 1.9
Right amygdala - Left inferior striatum	11.9 ± 5.1
Right amygdala - Left superior striatum	23.8 ± 2.4
Right amygdala - Left middle frontal gyrus (MFG)	−18.0 ± 3.1
Right_amygdala - Right supramarginal gyrus (SMG)	3.1 ± 2.7
Left inferior striatum - Left superior striatum	9.6 ± 1.2
Left inferior striatum - Right planum temporale (PT)	−10.4 ± 3.5
Left superior striatum - Right PT	11.2 ± 5.3
Left superior striatum - Left orbitofrontal cortex (OFC)	12.0 ± 3.0
Left superior striatum - Left MFG	13.5 ± 1.0
Left superior striatum - Right anterior cingulate cortex (ACC)	−30.3 ± 1.9
Left MFG - Left inferior frontal gyrus (IFG)	−4.3 ± 2.1
Right PT - Left MFG	−12.2 ± 2.2
Right PT - Left IFG	−3.9 ± 2.8
Right angular gyrus (AG) - Right SMG	1.8 ± 1.7
Left OFC - Right PT	18.6 ± 2.4
Left OFC - Right ACC	−10.4 ± 3.5
CBLN - Left MFG	13.9 ± 1.8

Finally, we constructed a logistic model that discriminated between FOG+ and FOG− patients, by using the same FCs selected by the correlational LASSO model. The logistic model yielded moderate (AUC = 0.64 ± 0.12) yet statistically significant performance with balanced accuracy (0.63 ± 0.12) and sensitivity (0.67 ± 0.16), using a binomial test (*p* < 0.05). However, the specificity (0.61 ± 0.20) did not reach statistical significance.

## 4 Discussion

We examined resting-state FCs that correlated with an index of FOG in 67 patients with PD. The results supported the previous proposal that the links across the emotional, cognitive, and motor networks underlie the pathophysiology of FOG (Ehgoetz Martens et al., 2018, Gilat et al., 2018). The NFOGQ scores were correlated with FC in the intra-BGN clusters and extra-CBLN clusters involving the FPN. We identified the amygdala in the exploratory (regionally unbiased) dual regression analysis whereas previous studies correlated the amygdala with FOG in hypothesis-driven VOI analyses (Gilat et al., 2018). Moreover, we constructed a statistical model using FCs that explained nearly 40 % of the inter-individual variance of FOG and that discriminated between patients with FOG or those without FOG. This model used information from the emotional network (amygdala and OFC), subcortical motor network (striatum and CBLN), and cognitive network (FPN). Importantly, the amygdala was the only node connecting to the subcortical motor and cortical cognitive networks in the present analysis. The amygdala has direct anatomical connections with the striatum and cognitive cortical regions including the prefrontal cortex (Fudge et al., 2002, Liu et al., 2013, Mcdonald, 1998, Russchen et al., 1985). These results suggest that the amygdala may have a pivotal role in the interactions across the emotional, cognitive-attentional, and subcortical motor networks, indicating it the involvement of the amygdala in the system-level impairment underlying FOG.

The amygdala is a core structure related to feelings of fear and anxiety (Adhikari et al., 2015, Butler et al., 2007, Duvarci and Pare, 2014, Phelps et al., 2001) and emotional response for negative visual stimuli (i.e. fearful face) (Kragel et al., 2021, Pessoa, 2017). Patients with FOG have higher anxiety than those without FOG (Ehgoetz Martens et al., 2016). Our results suggest that the increased FC in the amygdala and the putamen may reflect the anxiety state associated with FOG. A previous rsfcMRI study consistently showed increased positive FC between the amygdala and putamen along with enhanced anti-correlation between the amygdala and FPN in patients with FOG (Gilat et al., 2018). The role of the medial PFC and FPN in regulating the amygdala provide top-down control over attention/cognition and emotional responses (Kragel et al., 2021, Ochsner et al., 2012, Pessoa, 2017, Pessoa, 2008). The fact that the FC between the amygdala and cognitive network (FPN) was chosen by our statistical model may suggest a lack of top-down control of the FPN over the amygdala as a mechanism of FOG. Moreover, the amygdala function was implicated in subtypes of PD, one of which is closely related to FOG. Therefore, PD patients can be classified into postural instability/gait difficulty and tremor-dominant subtypes (Stebbins et al., 2013). FOG is more frequently observed in the postural instability/gait difficulty subtype, which has a lower amygdala volume compared with the tremor-dominant subtype (Forsaa et al., 2015, Rosenberg-Katz et al., 2016). Thus, converging evidence suggests a pivotal role of the amygdala in the emotional aspects of FOG. The role of the amygdala in FOG may be mediated by the periaqueductal gray matter (PAG) matter in the brainstem. Aspects of FOG resemble locomotor arrest, which occurs in response to external threats in rodents (defensive arrest). The neural circuits underlying defensive arrest include the amygdala, PAG, and medullary reticular formation. The pathway from the central amygdala to the ventrolateral PAG, disinhibits glutamatergic output from the ventrolateral PAG to the medullary reticular formation and is especially important in defensive arrest (Pernía-Andrade et al., 2021). Thus, the amygdala and PAG may be central to the provocation of FOG; unfortunately, the FC of a small structure such as the PAG could not be assessed using the method used in the current study.

The present BGN, which was used as the RSN template extracted by group-ICA for the analysis of the FOG correlation, extended into the amygdala and thalamus. The pathophysiology of gait disturbance in PD is thought to be related to abnormalities of the basal ganglia-thalamo-cortical and basal ganglia-brainstem systems (Hanakawa et al., 1999b, Hanakawa, 2006). Although the amygdala is considered the core of the emotional network in the limbic system, the amygdala and basal ganglia share substrates (ganglionic eminence of the telencephalon) at the prenatal developmental stage. Indeed, the amygdala and striatum have direct anatomical connections (Fudge et al., 2002, Russchen et al., 1985). Therefore, the amygdala and thalamus are closely connected to the basal ganglia. The BGN, amygdala, and thalamus likely constitute an extended BGN system, the abnormality of which is strongly implicated in the pathophysiology of FOG.

The NFOGQ-correlated BGN clusters were found within the BGN mask (intra-RSN), including the right amygdala. The FC between the right amygdala and striatum was included in the network related to FOG, but that of the left was not (Ehgoetz Martens et al., 2018). Hence, there could be asymmetry in the networks underlying FOG. Indeed, a majority of patients in this study had higher MDS- UPDRS-III scores on the left side, indicating that these results are likely explained by the right lateralized network abnormality.

The cerebellum, especially the vermis and fastigial nuclei, is important for the control of balance and locomotion (Morton and Bastian, 2004), and altered cerebellar functions may be related to FOG. The optical activation of afferents from the cerebellar fastigial nucleus at the ventrolateral PAG induced freezing in mice (Vaaga et al., 2020). Previously, intra- and extra-cerebellar connectivities were shown to be correlated with FOG (Bharti et al., 2019a, Fasano et al., 2017, Fling et al., 2014). Furthermore, enhanced activity in the cerebellum, premotor areas, ACC, and posterior parietal cortex was shown during visually-guided gait, which can often overcome FOG in PD (Hanakawa et al., 1999a). The fronto-parietal cognitive network was shown to have extra-network connectivity to the CLBN. This finding probably reflects the connection between the cerebellum and fronto-parietal cognitive network, which is essential for executive control (Miller, 2000). The cerebellum connects to the prefrontal cortex in non-human primates (Middleton and Strick, 2001) and humans (Buckner et al., 2011). Patients with FOG are characterized by cognitive dysfunctions (Brugger et al., 2015, Naismith et al., 2010), including impairment of attentional “set-shifting” (Smulders et al., 2015). Notably, a *meta*-analysis reported that Crus1, present in the CBLN, was activated during executive function tasks (Stoodley and Schmahmann, 2009). Thus, it is reasonable to assume that the fronto-parietal cognitive network is involved, at least in part, in the pathophysiology of FOG in relation to the cognitive functions of the cerebellum. In particular, a positive correlation between the NFOGQ scores and cerebellar-cortical cognitive network FC may reflect problems in attentional switching underlying FOG.

To our knowledge, this is the first study to construct statistical models to predict the severity of FOG from FCs. The employed FCs, which were derived from a dual regression analysis, reflected the interactions across the emotional, cognitive-attentional, and subcortical motor networks. LASSO regression has the advantage of introducing sparsity using the L1 norm to reduce the dimensions of the features. Moreover, a logistic model using the same FCs predicted the presence of FOG. These two novel analyses confirmed that FCs representing the interactions across the emotional, cognitive, and subcortical motor networks reflect the system-level impairment underlying FOG. In the future, such statistical models using FCs may improve the diagnosis and evaluation of FOG in clinical settings.

This study had some limitations. First, we obtained fMRI data only in the medication “on” state. Acquiring fMRI from PD patients under medication has practical merits; PD patients are easier to study when on medication because they have fewer involuntary movements (e.g. tremor). By contrast, dopaminergic treatment potentially influences FC, and this confounding effect can make the interpretation of FC difficult (Tahmasian et al., 2015). Second, we obtained resting-state fMRI data only, not task-fMRI. Task-fMRI studies should allow us to examine brain activity during the appearance of FOG-related phenomena whereas resting-state fMRI does not. However, both task-fMRI (Ehgoetz Martens et al., 2018) and resting-state fMRI (Gilat et al., 2018) study showed similar motor, emotional, and cognitive network associations. In view of these points, a future study comparing task fMRI and resting-state fMRI data in both ''on'' and ''off'' states should provide more comprehensive understanding of the mechanisms underlying FOG. Third, the present study was based on a single cohort. The inclusion of more participants from independent cohorts would help create a machine learning model using FCs. Moreover, we included both FOG+ and FOG− patients in the model building stage; especially when the NFOGQ score was 0, the distribution of NFOGQ score could be skewed. Future studies should include>100 participants, especially freezers, to create a prediction model, which can be generalized to a validation cohort.

## 5 Conclusions

We confirmed the links across the emotional, cognitive-attentional, and subcortical motor networks underlying the pathophysiology of FOG, by combining rsfMRI data and machine learning-based statistical models. Particularly, the amygdala emerged as a key node that connected the subcortical motor (BGN and CBLN) and cognitive (FPN) networks. Future refinement of the machine learning methodology based on the emotional, cognitive-attentional, and subcortical motor networks FCs may improve the diagnosis and evaluation of FOG in clinical settings.

## CRediT authorship contribution statement

**Hiroki Togo:** Conceptualization, Methodology, Validation, Formal analysis, Investigation, Writing – original draft, Writing – review & editing, Visualization. **Tatsuhiro Nakamura:** Conceptualization, Methodology, Validation, Formal analysis, Investigation, Writing – review & editing, Visualization. **Noritaka Wakasugi:** Conceptualization, Methodology, Investigation, Writing – review & editing. **Yuji Takahashi:** Conceptualization, Writing – review & editing, Project administration. **Takashi Hanakawa:** Conceptualization, Methodology, Validation, Formal analysis, Investigation, Writing – original draft, Writing – review & editing, Project administration, Funding acquisition.

## Declaration of Competing Interest

The authors declare that they have no known competing financial interests or personal relationships that could have appeared to influence the work reported in this paper.

## Data Availability

Data will be made available on reasonable request.
