# Supplementary material for: Interactions across emotional, cognitive and subcortical motor networks underlying freezing of gait
Source: Neuroimage Clin. 2023 Feb 2;37:103342. doi: 10.1016/j.nicl.2023.103342 (PMC9932566; doi:10.1016/j.nicl.2023.103342)
Supplement: Supplementary data 1 [file mmc1.docx]

**Supplementary Materials**

**Sensitivity to FOG-related alterations to network organization**

We also performed group-spatial ICA on the rsfcMRI data only from the 67 PDs to examine how the difference in the RSNs affected sensitivity to alterations of FOG-related network disorganization. The concatenated rsfcMRI volumes were also decomposed into 40 spatial components. We visually investigated all ICA components and identified the three ICA components of interest (i.e., BGN, CBLN, and SMN) (Supplemental Fig. 1). We were able to retrieve similar RNSs in both analyses, suggesting that the general pattern of the BGN, CBLN, and SMNs were shared across the PD patients and controls. We performed the dual regression correlation between the NFOGQ scores and FC related to the three RSNs of interest (BGN, CBLN, and SMN) by using the spatial maps derived from the PD patients only. However, we did not find the significant clusters correlated with the NFOGQ scores in this analysis as opposed to the analysis using the spatial maps derived from both PD patients and controls. This discrepancy indicated the importance of the spatial maps used for the dual regression. We suspect that the extraction of spatial maps from both groups made the RSNs more reliable because of: 1) a greater number of participants contributing to the spatial maps and/or 2) a more generalized spatio-temporal pattern that can be separated into independent RSNs by group ICA.

**
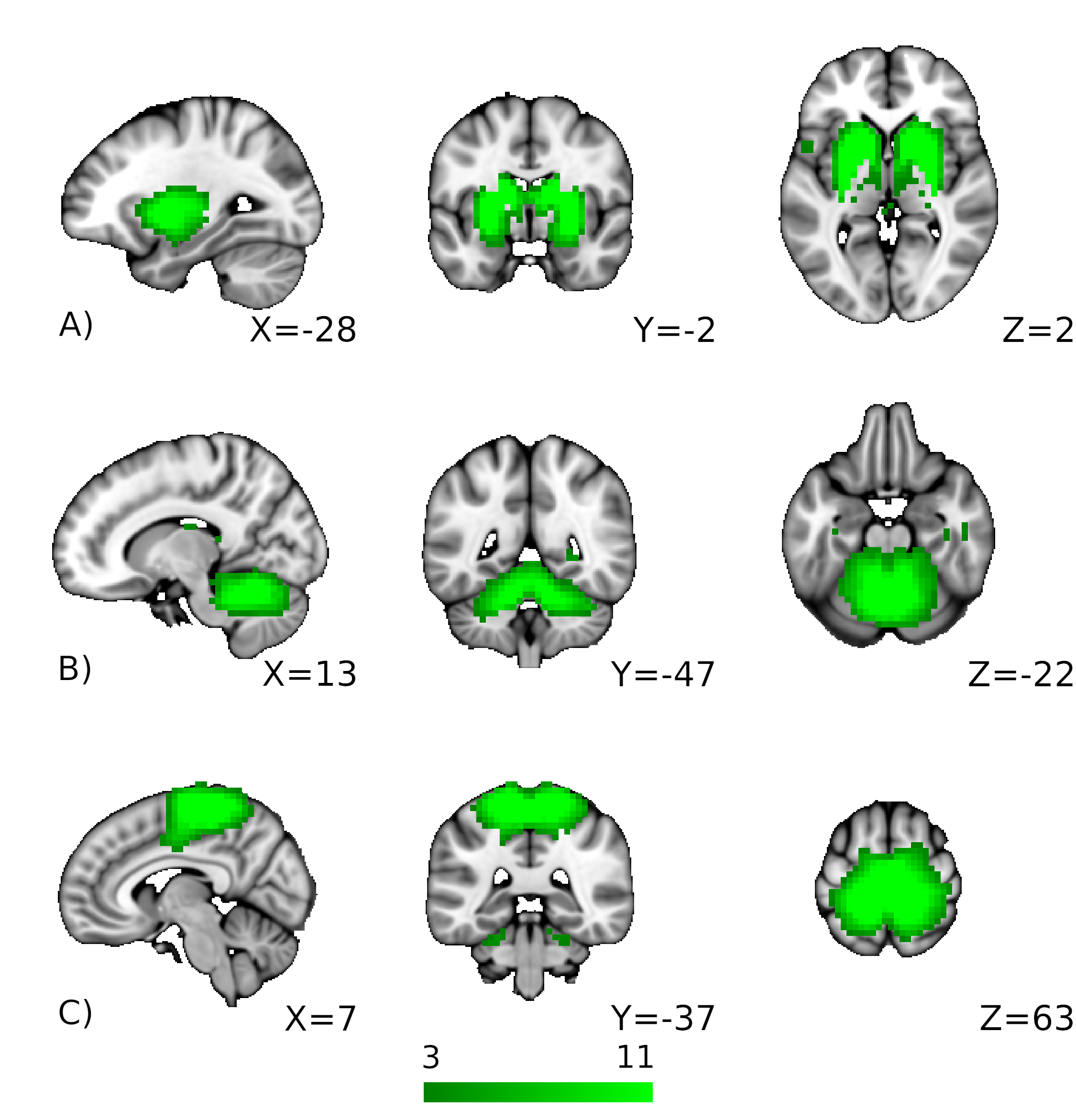
**

**Supplementary Fig. 1.** Three resting-state networks of interest (green) created only from the Parkinson’s disease (PD) patients. A) Basal ganglia network (BGN), B) cerebellar network (CBLN), and C) sensorimotor network (SMN). The color bar indicates z-values thresholded at the default value (z >3).

**Functional connectivity analysis with age and sex as covariates**

The correlation analysis of FC with the NFOG score revealed almost identical findings with (supplementary Figure 2) and without (Fig. 2) using age and sex as covariates.  **
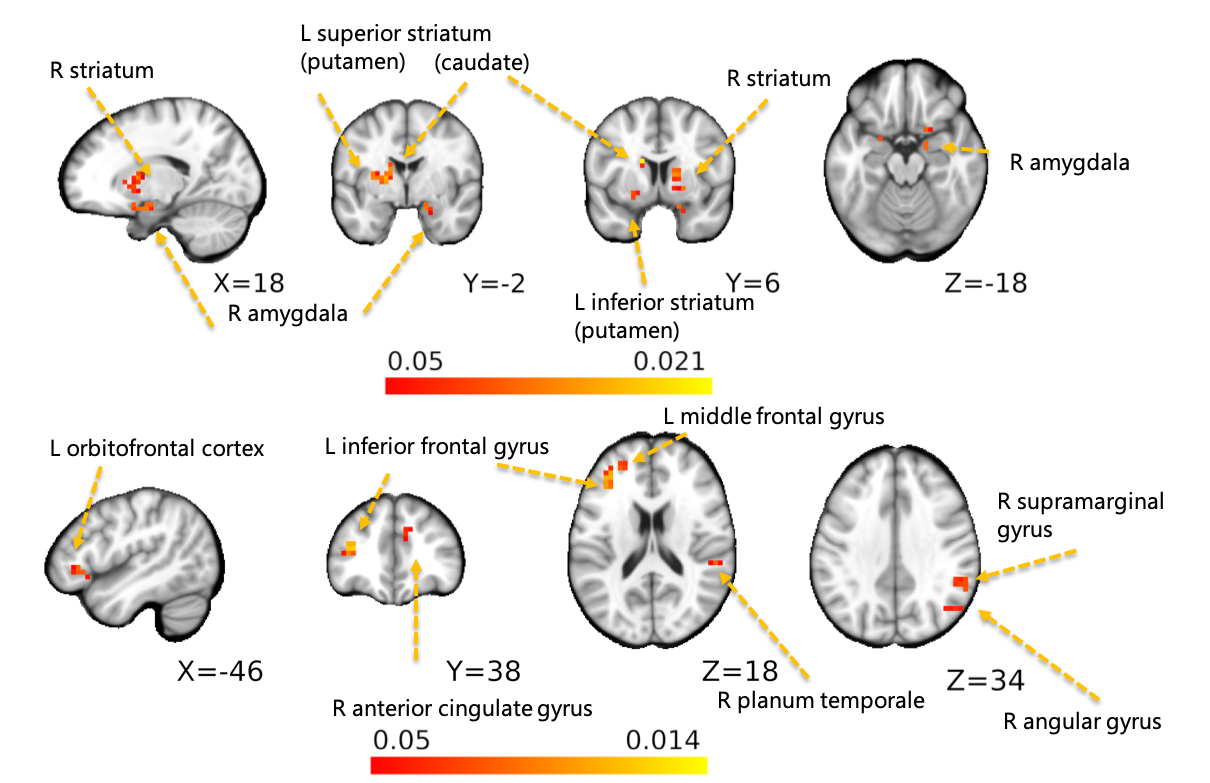
 Supplementary Fig. 2.** The functional connectivity analysis with age and sex as covariates. The hot color scaled areas indicate a correlation of freezing severity with functional connectivity of the basal ganglia network (top) and the cerebellar network (bottom) (corrected *p* < 0.05).
